# Endoscopic Extended Sinus Surgery for Patients with Severe Chronic Rhinosinusitis with Nasal Polyps, the Choice of Mucoplasty: A Systematic Review

**DOI:** 10.1007/s11882-023-01113-x

**Published:** 2023-11-22

**Authors:** Daniel Martin-Jimenez, Ramon Moreno-Luna, Alfonso Cuvillo, Jaime Gonzalez-Garcia, Juan Maza-Solano, Serafin Sanchez-Gomez

**Affiliations:** 1 Department of Otolaryngology, Head and Neck Surgery, Rhinology Unit, Virgen Macarena University Hospital, Doctor Fedriani Av. 3, Seville, 41009 Spain; 2 Unidad de Rinología y Asma, UGC ORL, Hospital Universitaro De Jerez, Instituto De Investigación Biomedica De Cadiz (INIBICA), Jerez, 11407 Spain; 3 Department of Surgery, University of Seville, C. San Fernando, 4, Sevilla, 41004 Spain

**Keywords:** Nasal polyps, Chronic rhinosinusitis, Nasal surgical procedures, Type 2 inflammation, Reboot surgery, Mucoplasty

## Abstract

**Purpose of Review:**

The advances in the knowledge of the molecular basis of the inflammatory response in chronic rhinosinusitis with nasal polyps (CRSwNP) have led the management of these patients towards personalized and precision medicine. Surgery has been positioned as a suitable alternative in patients who do not achieve control with appropriate medical treatment, but polypoid recurrences remain a constraint. The emergence of new surgical approaches based on patient phenotyping and the poor disease control associated with type 2 inflammatory phenotype makes it necessary to review the role of personalized and precision surgery in managing the disease.

**Recent Findings:**

Surgical approaches based on wide resection of bony sinus structures and the treatment of mucosa lining the sinonasal cavity have been analyzed and compared with other techniques and seem to offer more favorable surgical outcomes and improved quality of life (QoL), in addition to lower relapse rates. The innovations with new complementary surgical techniques, such as reboot surgery adding an extended autologous mucosal graft from the nasal floor (mucoplasty), may benefit endoscopic and QoL outcomes in the most severe CRSwNP patients with type 2 phenotype.

**Summary:**

Using bilateral endonasal mucoplasty as a complementary technique to reboot surgery is a suitable technical choice that has improved short- and medium-term QoL and endoscopic outcomes for patients with severe CRSwNP. These results are likely due to a combination of the extension of reboot and the inherent inflammatory and healing properties of mucoplasty. We propose this technique as a valuable surgical resource, although more robust clinical studies are needed to evaluate its long-term benefits comprehensively.

**Supplementary Information:**

The online version contains supplementary material available at 10.1007/s11882-023-01113-x.

## Introduction

Chronic rhinosinusitis with nasal polyps (CRSwNP) is an inflammatory sinonasal disease that is estimated to affect between 0.5 and 4.5% of the general population, with a proven significant impact on patient’s quality of life (QoL) and a high economic cost to healthcare systems [1]. Initial treatment involves nasal rinses and intranasal steroids, leaving systemic corticosteroids for the exacerbations, constituting the so-called appropriate medical treatment (AMT) [2–4]. In patients whose AMT is insufficient to achieve symptom control, endoscopic sinus surgery (ESS) has been proposed as a suitable alternative [5]. However, there is no consensus on the optimal surgical strategy in CRSwNP, and polyp recurrences remain a significant limitation of this therapeutic approach [6]. Moreover, since the guideline’s recommendations on the management of CRSwNP are constantly changing, surgery has become a fundamental criterion for indicating the recent treatment option with biological therapies [7].

Comorbidities, such as late-onset asthma and nonsteroidal anti-inflammatory drugs-exacerbated respiratory disease (N-ERD), or a history of re-interventions, are associated with poorer disease control and often account for the failure of the medical and surgical treatments [8]. Furthermore, the increasing knowledge of the underlying mechanisms that produce mucosal inflammation in CRSwNP (the mucosal concept) [9•] and the description of several biomarkers, such as immunoglobulin E (IgE), eosinophilic cationic protein (ECP), or interleukins (IL) 4, 5, and 13, have led the description of subjacent inflammatory endotypes and consequent phenotypes and allow the application of the precision medicine paradigm in the management of CRSwNP patients [10, 11]. In this sense, the advances in the knowledge of the type 2 endotype have allowed the development of new and more extended surgical approaches to manage these disease’s severe and recalcitrant phenotypes [12, 13]. This change in the CRSwNP paradigm has meant that techniques such as full-house or reboot surgery, based on wide resection of bony sinus structures and the treatment of mucosa lining the sinonasal cavity, have been analyzed and compared with other techniques to assess if they are associated with more favorable surgical outcomes and improved QoL, in addition to lower relapse rates [14, 15••].

Our group home-grown classification of the different techniques of ESS published to date, including a thorough description of their characteristics attending to the surgical modification performed on the anatomic structures of the sinonasal cavity, is shown in supplementary files (Table S1).

We have also shown that an extended autologous mucosal graft from the nasal floor positioned in the ethmoidal roof (mucoplasty) associated with reboot surgery may provide an added benefit to endoscopic and QoL outcomes in CRSwNP patients with type 2 phenotype by improving not only the healing but also the post-surgical inflammatory pattern with a regenerative role [16••].

The objective of our study is to systematically review the expected benefit of extended endoscopic sinus surgery (EESS) versus other approaches and the role of mucoplasty as a conceptually regenerative option in the surgical management of severe CRSwNP patients.

## Material and Methods

This systematic review has been reported following the recommendations of the Preferred Reporting Items for Systematic Reviews and Meta-Analyses (PRISMA) [17] (supplementary files—Table S2 contains the PRISMA checklist fulfilled). No review protocol was registered for this study.

### Research Question

We aimed to answer the following research question: What are the advantages of adding mucoplasty to reboot surgery in treating severe chronic rhinosinusitis with nasal polyp patients versus the medical or surgical standard of care?

### Search Strategy

The search strategy was designed using the PICOTs framework:

- Participants: Severe CRSwNP patients older than 18, non–responders to adequate medical treatment who undergo ESS.
- Intervention: Eligible interventions included extended ESS (e.g., nasalization, complete, radical, full-house, reboot, reboot) with or without mucoplasty.
- Comparators: Standard of care of medical or surgical treatment, following international guidelines [2, 3].
- Outcomes: Quality of life (QoL) and/or symptom scale improvement, changes in nasal endoscopy scores and/or computed tomography (CT) scan scores.
- Timing and Setting: With no limitations.

According to PRISMA statement recommendations, we searched in the following databases: PubMed, The Cochrane Library for Cochrane Reviews, Embase via Elsevier, Web of Science, and Scopus from inception until May 2023. The search strategy is described in supplementary files (Table S3). To supplement the database search, we manually checked the reference lists of the included studies, performed a backward citation analysis, and completed a forward citation analysis.

### Eligibility Criteria

Inclusion criteria for studies to select were articles written in English, which type of design has been clinical trials, cohort studies, case–control studies, cross-sectional studies, or case-series studies published in peer-reviewed journals.

Exclusion criteria were studies dealing with localized and/or systemic CRSwNP, bilateral inflammatory disease but without nasal polyps, unilateral surgical approaches, or CRSwNP in treatment with monoclonal antibodies during the study period.

### Study Extraction, Categorization, and Analysis

Screening by title and abstract was conducted by three authors (DMJ, RML, JMS) independently. After title and abstract screening and discard, full texts were retrieved for the remaining articles. Two authors (DMJ, RML) reviewed the full texts against the inclusion criteria. Discrepancies were resolved by consensus.

A standardized form (initially piloted on six included studies) was used for data extraction of characteristics of studies, outcomes, and risk of bias. Data extraction was conducted by two authors (DMJ, RML). Extracted variables encompassed: sample size, age, gender, comorbidities such as asthma, NSAID-exacerbated respiratory disease, or proven allergic sensitization, type of surgery, and the primary outcomes in terms of QoL (Sinonasal Outcomes Test–22), symptom severity (visual analogue scale (VAS), total nasal symptom score (TSS)), nasal endoscopy scores (nasal polyp score (NPS), Meltzer scale, total polyp score (TPS), modified Lund-Kennedy (MLK) scale), or sinus CT scan (Lund Mackay (LM) scale).

### Assessment of Study Quality and Risk of Bias

Studies selected for the systematic review were assessed about quality using the Oxford Centre for Evidence-Based Medicine Levels of Evidence [18]. The risk of bias was assessed using the Quality Assessment of case series studies checklist from the National Institute for Health and Clinical Excellence (Fig. 2 and Supplementary Table S4) [19]. Three authors (DMJ, RML, JMS) independently performed the evaluation, and consensus solved discrepancies.

### Statistical Analysis

Meta-analyses were not possible due to the outcomes’ high heterogeneity between studies, so only qualitative analysis was performed. Three authors (DMJ, RML, JMS) discussed the magnitude and relevance of the effects assessed for the qualitative analysis. Discrepancies were solved by consensus, and these discussions were reflected in the discussion section.

Superior postoperative outcomes for severe CRSwNP patients were considered when a more considerable improvement in the QoL or symptoms scales was shown (SNOT-22, VAS, or other analyzed scales), as well as when decreases in the values of endoscopic and radiological scales were observed after surgery (NPS, Meltzer scale, TPS, MLK scale, and LM score). Other features of interest in the qualitative evaluation of the studies were tissue and peripheral blood cell counts, measurements of tissue biomarkers, the control of lower airways comorbidities (asthma or N-ERD), the rates of polypoid recurrences, the need for revision surgery, and changes in the assessment of smell.

## Results

**Fig. 1:**
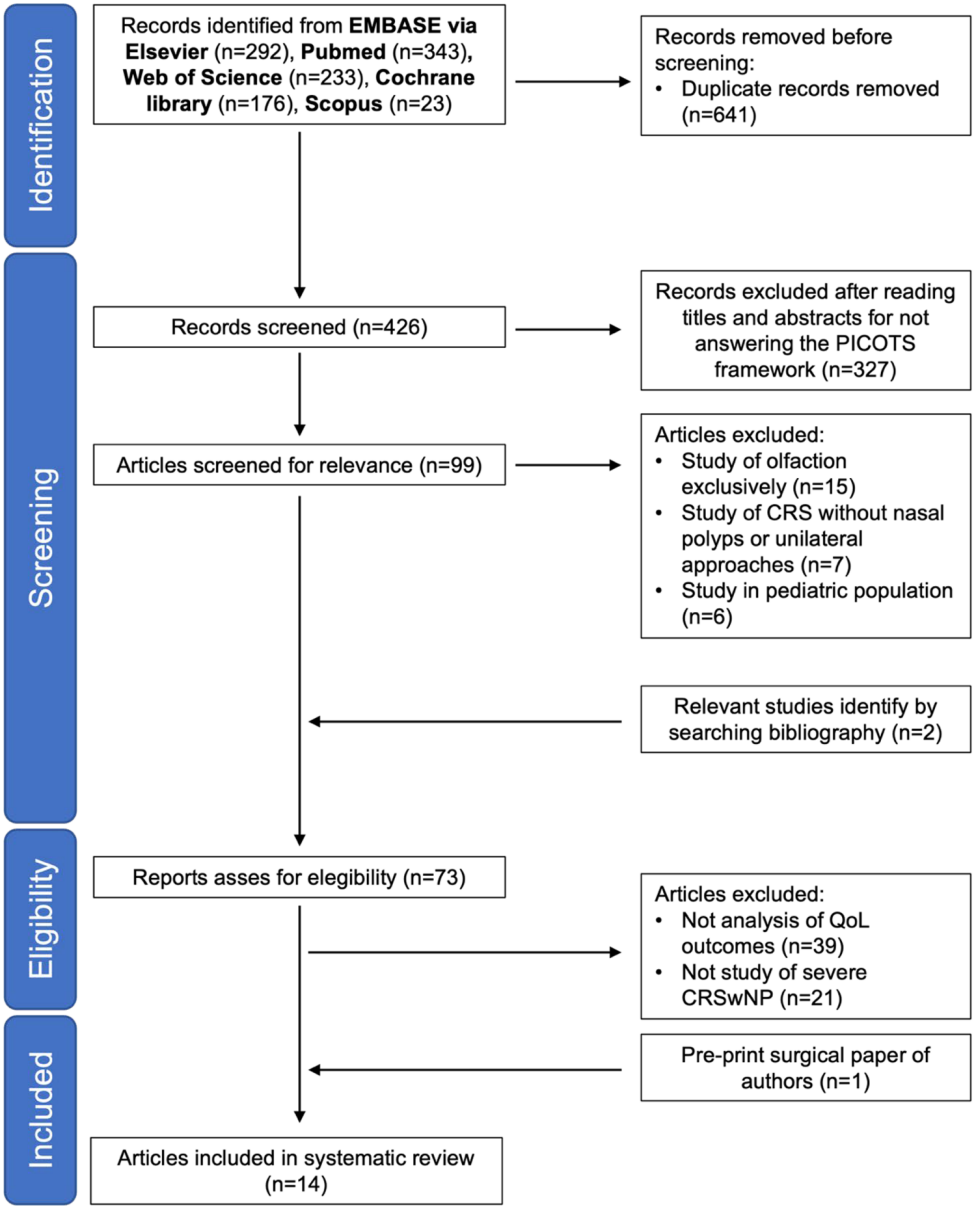
PRISMA flow diagram. Abbreviations: CRS, chronic rhinosinusitis; CRSwNP, chronic rhinosinusitis with nasal polyps; QoL, quality of life

The bibliographic search for the systematic review was performed on May 23, 2023, and identified 1067 potentially relevant studies. After removing duplicates and applying the inclusion and exclusion criteria, 13 articles and a study yet to be published from our group were included in qualitative synthesis for the data extraction**.** The selection process was recorded in sufficient detail to complete a PRISMA flow diagram (see Fig. 1).

All included articles had relevance to the subject of this review. Two were non-randomized controlled clinical trials, one a prospective case–control study, four retrospective case–control, three prospective case series, and the remaining four papers were retrospective case series.

**Fig. 2:**
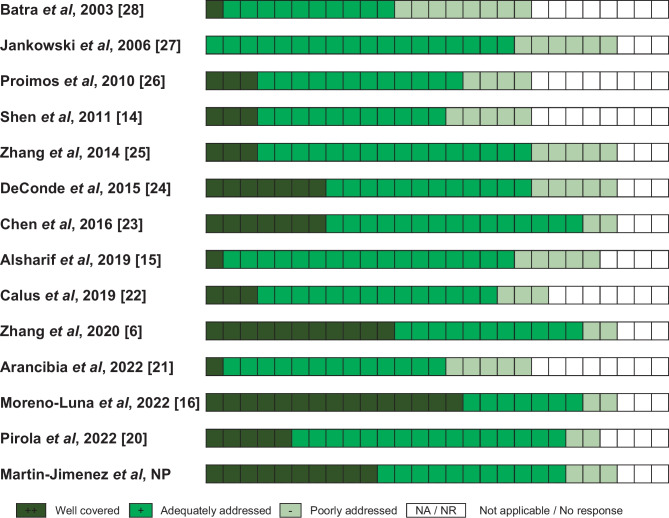
Quality Assessment of case series studies checklist from the National Institute for Health and Clinical Excellence (Appendix F) applied to this systematic review. The color graphic represents the answer to each checklist question from 1.1 to 5.2 (27 questions); the specific answers are shown in Supplementary Table S4

Table 1 describes the included studies’ quality characteristics and features in terms of follow-up period, sample size, age of the sample, sex, asthma and N-ERD prevalence, proven allergic sensitization, and history of previous ESS. Figure 2 summarizes the assessment risk of bias using the Quality Assessment of case series studies checklist from the National Institute for Health and Clinical Excellence (published as Appendix F).

**Table 1:** Summary table of demographics characteristics and biomarkers distribution of the articles included in this review

Authorship	Year	Type and evidence of study	Follow-up	Sample size	Age (years) Mean ± SD or range	Sex (M, F)	Asthmatics	N-ERD patients	Proven allergic sensitization	History of previous ESS
**Martin-Jimenez et al**	data not published	Case–control 3b^b^	2 years	274	50.3 ± 13.4	66.1% M; 33.9% F	135 (49.3%)	59 (21.5%)	145 (52.9%)	90 (32.8%)
**Pirola et al.** [20]	2023	Case-series 4^b^	2 years	30	50.5 ± 12.5 (range 18–74)	46.7% M; 53.3% F	22 (73.3%)	6 (20.0%)	25 (83.3%)	30 (100%)
**Arancibia et al.** [21]	2022	Prospective case-series 4^b^	5 and 12 years	76	46.8 ± 13	60.5% M; 39.5% F	41 (53.9%)	23 (30.3%)	NR	15 (19.7%)
**Moreno-Luna et al.** [16••]	2022	Non-randomized controlled clinical trial 2b^b^	1 year	64	- Mucoplasty group: 50.5 ± 11.4 - RESS group: 49.3 ± 14.5	57.8% M; 42.2% F	43 (67.2%)	19 (29.7%)	30 (46.9%)	32 (50.0%)
**Zhang et al.** [6]	2020	Non-randomized controlled clinical trial 2b^b^	1, 3, and 5 years	81	- RESS + DRAF3 group: 47.30 ± 11.03 - EESS group: 41.37 ± 12.76 - FESS group: 44.56 ± 11.23	59.3% M; 40.7% F	81 (100%)	24 (29.6%)	26 (32.1%)	81 (100%)
**Alsharif et al.** [15••]	2019	Case–control 3b^b^	2 and a half years	50	- Full RESS group: 39.5 ± 11.9 - Partial RESS group: 52.3 ± 14.0 - Non-RESS group: 48.7 ± 13.1	68.0% M; 32.0% F	50 (100%)	4 (8.0%)	21 (42.0%)	33 (66.0%)
**Calus et al.** [22]	2019	Prospective case-series 4^b^	6 and 12 years	47	49 (range 37–58)	70.2% M; 29.8% F	18 (38.3%)	11 (23.4%)	24 (51.1%)	25 (53.2%)
**Chen et al.** [23]	2016	Case–control 3b^b^	1 year	47	- EESS group: 42.65 ± 8.86 - FESS group: 48.82 ± 11.15	54.8% M; 45.2% F	42 (89.4%)	NR	20 (42.6%)	10 (21.3%)
**DeConde et al.** [24]	2015	Prospective case–control 2b^b^	13 months on average	311	- Complete surgery group: 52.4 ± 15.3 - Targeted surgery group: 52.6 ± 14.7	47.9% M; 52.1% F	115 (37.0%)	26 (8.4%)	121 (38.9%)	162 (52.1%)
**Zhang et al.** [25]^**a**^	2014	Case-series 4^b^	1, 3, and 6 months	376	48.43 ± 13.34	62.5% M; 37.5% F	151 (40.2%)	28 (7.4%)	170 (45.2%)	156 (41.5%)
**Shen et al.** [14]	2011	Case-series 4^b^	6, 12, and 24 months	21	42.6 ± NR	57.1% M; 42.9% F	1 (4.8%)	NR	7 (33.3%)	21 (100%)
**Proimos et al.** [26]	2010	Prospective case-series 4^b^	6 months and 1 year	86	46.9 ± 14.9	38.4% M; 61.6% F	86 (100%)	NR	NR	NR
**Jankowski et al.** [27]^**a**^	2006	Case–control 3b^b^	5 years	76	- Nasalization group: 47.2 (range 28–71) - Functional ethmoidectomy group 44.0 (range 26–65)	69.8% M; 30.2% F	14 (18.4%)	15 (19.7%)	NR	39 (51.3%)
**Batra et al.** [28]	2003	Case-series 4^b^	1 year	17	51 (range 31–80)	58.8% M; 41.2% F	17 (100%)	9 (51.9%)	NR	NR

*EESS* extended endoscopic sinus surgery. *F* female. *FESS* functional endoscopic sinus surgery. *M* male. *NR* not reported. *RESS* reboot endoscopic sinus surgery

^**a**^Only patients who complete the follow-up were counted

^b^Level of evidence was evaluated according to the Oxford Centre for Evidence-Based Medicine Levels

Table 2 summarizes the main findings of the qualitative analysis of included articles in this systematic review, including the outcomes reported per article. EESS reflected better results in the QoL questionnaires, polypoid growth scales, and better radiological imaging measures than functional surgeries. Recurrence rates, disease-free time, control of comorbidities, and olfaction study outcomes were also higher associated with more extended approaches. Disparate results were found in the analysis of molecular biomarkers, cell counts, and endoscopic findings of edema and nasal discharge.

**Table 2:** Summary table of the main characteristics and results of the articles included in this review

Authorship	Year	Groups distribution in sample	Clinical outcomes (QoL and symptoms scale)	Endoscopic and radiological post-surgical scores	Other outcomes
**Martin-Jimenez et al.**	data not published	274 patients. Two surgical groups: - EESS group (*n* = 111) - FESS group (*n* = 163)	- EESS improved 39.2 points in SNOT-22, while FESS was 27.6 units - Through a multivariate analysis: EESS improved SNOT-22 in 14.8 units, against FESS, and its OR = 6.5 (95%CI: [1.70, 24.84]) to achieve MCID	Baseline NPS, MLK scale and LM score data were collected but postoperative outcomes were not analyzed	- Lower re-intervention rate associated with EESS, without further complications - Worse self-reported baseline QoL associated with greater postoperative improvement in linear and logistic multivariate regression models
**Pirola et al.** [20]	2023	Patients underwent partial reboot surgery with previous ESS (*n* = 30)	- Partial reboot improved 38.16 units in SNOT-22 - Every VAS record showed significant improvement	Baseline NPS and LM are collected but postoperative outcomes are not analyzed	- Recurrence-free survival showed differences in favor of reboot surgery versus conventional ESS, being significant at each time point - No systemic steroids courses were needed after partial reboot in any patient
**Arancibia et al.** [21]	2022	Patients underwent EESS (*n* = 76)	- SF-36 physical summary increased by 9.4 points at 5 years and was maintained at 12 years of follow-up - The median difference in TSS was 7 in both periods analyzed	- An improvement of 6 points in NPS was recorded at 12 years - LM score decreased 8 units at 12 years follow-up (Me [IQR] = 12 [9.2–15]) - MLK scale was not recorded	- 21.9% required revision ESS - An increase in all tests of olfactometry BAST-24 was obtained
**Moreno-Luna et al.** [16••]	2022	64 patients. Two surgical groups: - RESS group (*n* = 47) - RESS with bilateral mucoplasty group (*n* = 17)	- RESS with bilateral mucoplasty improved by 62.7 points in SNOT-22. Isolated RESS improvement was 43.2 units - Through a multivariate analysis: RESS plus bilateral mucoplasty improved SNOT-22 in 22.6 units against patients who underwent reboot surgery only	- Better results in NPS and LM score were observed after the use of bilateral mucoplasty - No postoperative differences were found in MLK scale	- A rate of revision surgery equal to 0% was described for bilateral mucoplasty group - No major complications were reported - Poorer outcomes were associated with history of previous ESS
**Zhang et al.** [6]	2020	81 patients. Three surgical groups: - FESS group (*n* = 27) - RESS group (*n* = 27) - RESS + DRAF 3 group (*n* = 27)	- RESS increased SNOT-22 at 1 year, more than FESS. No differences were found at other follow-up points in time - RESS and RESS + DRAF 3 reported better improvement ratios in rhinorrhea and smell throughout the monitoring	- No differences in MLK scale were reported among groups - NPS and LM score were not analyzed	- At 3 or 5 years postoperatively, the recurrence rate ranged about 95% and was not significantly different among groups - No patient had revision surgery within 1-year post-surgery, but more patients in the FESS group required a new surgery by 3 to 5 years - There was no difference among the groups regarding clinical control of asthma
**Alsharif et al.** [15••]	2019	50 patients. Three surgical groups: - FESS group (*n* = 20) - Partial RESS group (*n* = 18) - RESS group (*n* = 12)	- Postoperative SNOT-22 was statistically significant lower in RESS group versus de FESS group - No differences in VAS were reported among groups after surgery	- NPS, MLK scale and LM score were not analyzed	- RESS group had significantly reduced relapse rates (8%) compared to patients undergoing classical FESS approach (45%) - Lower recurrences and longer disease-free time were reported in RESS group
**Calus et al.** [22]	2019	Patients underwent primary or revision EESS (*n* = 47)	- A scale graded as no, mild, moderate or severe, showed significant long-term improvement in nasal obstruction and smell disorder - No validated QoL questionnaire was studied	- Davos polyps score showed a statistically significant decrease over the 12-year period - MLK scale and LM score were not analyzed	- Logistic regression showed a higher risk of recurrence of nasal polyps in allergic patients (OR = 4.5, 95% CI: [0.78 to 26.1]), but not in asthmatics or N-ERD - Tissular IL-5, ECP and IgE levels were not associated with an increase of recurrence
**Chen et al.** [23]	2016	47 asthmatics patients. Two surgical groups: - EESS group (*n* = 23) - FESS group (*n* = 24)	- VAS scores were more improved in EESS group, with statistically significant differences with FESS groups only in olfaction	- E-scale to define endoscopic appearance of the sinus and olfactory clefts, after ESS, showed a better endoscopic outcome in EESS group - No postoperative differences were found in MLK scale	- No major complications were observed in both groups during surgery and at the 1-year follow-up period - There were no significant differences of change in ACT scores and pulmonary function indexes (FEV_1_, PEF, FEV_1_:FVC)
**DeConde et al.** [24]	2015	311 patients. Two surgical groups: - Complete surgery group (*n* = 147) - Targeted surgery group (*n* = 164)	- Complete ESS improved 28.1 points in SNOT-22, while targeted was 21.9 units - Subjects undergoing complete ESS also experienced greater absolute increase on SNOT-22 rhinologic and extra-rhinologic symptom domain scores - Through a multivariate analysis: complete ESS reduced SNOT-22 in 5.9 units, against FESS	- Better outcomes in MLK scale were observed in patients undergoing complete ESS, with no significant differences in multivariate analysis - Baseline LM score data were collected but postoperative outcomes were not analyzed	Olfaction (measured by B-SIT score) showed better post-operative outcomes after complete ESS, but no significant differences between approaches were found in multivariate analysis
**Zhang et al.** [25]	2014	Patients underwent FESS with CRS plus asthma, nasal polyps or both (*n* = 376)	FESS reduced 22.3 units in SNOT-22, at 6-months follow-up in CRSwNP asthmatic patients. Score progressively worsen in successive visits, while maintaining differences with baseline values	NPS, MLK scale and LM score were not analyzed	Worse self-reported baseline QoL correlated with greater postoperative improvement
**Shen et al.** [14]	2011	Patients underwent FESS with previous ESS (*n* = 21)	A non-validated Likert grading scale system, named Modified Patient Response Score, showed assessment showed rhinorrhea, nasal obstruction, postnasal dripping, smell sensation and headache was observed	- A significant decrease in muco-pus and mucosal swelling findings was observed at each follow-up time point - A statistically significant decrease was reported for LM score	Subgroup analysis by presence or absence of preoperative nasal polyposis or allergy revealed no outcome difference about LM score, muco-pus, mucosal swelling or PRS
**Proimos et al.** [26]	2010	Asthmatics patients underwent FESS (*n* = 86)	- The mean decrease from baseline to 12-months was − 0.9 for SNOT-22 and − 1.6 for SNOT-5 - A decrease in all VAS score values was reported at each time point	- A significant decrease in frequency of patients with nasal polyps, oedema and nasal discharge was observed - No validated scales for endoscopy o CT-scan were studied	- Nasal inspiratory peak flow was significantly increased - FVC, FEV_1_ and PEF significantly increased from baseline to six and 12-months - The proportion of hospitalizations, use of oral steroids and bronchodilators was significantly reduced
**Jankowski et al.** [27]	2006	76 patients. Two surgical groups: - Nasalization group (*n* = 39) - Functional ethmoidectomy group (*n* = 37)	- Global VAS improved 8.4 units in nasalization group, against 5.7 in ethmoidectomy group	A pre-defined endoscopic scale and CT-scan scores showed better post-operative outcomes in nasalization group	- Recurrence rate was 22.7% in the nasalization group, and 58.3% in the ethmoidectomy group
**Batra et al.** [28]	2003	Patients underwent polypectomy and FESS (Messerklinger technique) (*n* = 17)	- Sinonasal symptomatology measured through a subjective non-validated scale showed statistically significant differences after ESS - No validated QoL questionnaire was studied	- An improvement in post-operative LM scores were found - Endoscopic scores (NPS or MLK scale) were not analyzed	- A significant reduction in steroid usage was detected - Changes in FEV_1_ determination were observed after ESS

*BAST-24* Barcelona smell test 24, *ECP* eosinophilic cationic protein, *EESS* extended endoscopic sinus surgery, *ESS* endoscopic sinus surgery, *FESS* functional endoscopic sinus surgery, *IgE* immunoglobulin E, *IL* interleukin, *LM* Lund-Mackay score, *MCID* minimal clinically important difference, *MLK* modified Lund-Kennedy, *N-ERD* NSAIDs exacerbated respiratory disease, *NPS* nasal polyps score, *QoL* quality of life, *RESS* reboot endoscopic sinus surgery, *SF-36* Short Form-36 Health Survey, *SNOT-22* Sinonasal Outcomes Test 22, *TSS* total symptoms score, *VAS* visual analogue scale, *ACT* asthma control test, *B-SIT* brief smell identification test, *FEV1* forced expiratory volume in 1 s, *FVC* forced vital capacity, *PEF* peak expiratory flow, *PRS* patient response score, *QoL* quality of life, *SNOT-5* Sinonasal Outcomes Test 5

## Discussion

This systematic review of studies investigating the role of EESS and mucoplasty added to reboot surgery has retrieved 14 studies, all with promising results for extended ESS for the treatment of severe CRSwNP patients, but also concluding that more high-quality surgical studies are needed to accurately define these results and the benefit of adding mucoplasty. Despite this, incorporating mucoplasty as a regenerative approach may be associated with better outcomes than other EESS in managing severe CRSwNP patients by improving healing and post-surgical quality of life versus reboot surgery alone.

Selecting suitable surgical treatment for severe CRSwNP remains controversial in patients who do not achieve disease control with appropriate medical treatment. A wide range of ESS techniques, functional/targeted [26, 29] or extended surgeries [15••, 24, 27], with different extensions and nuances, has redefined their selves focused on advances in the pathophysiological understanding of this disease (Table S1 supplementary files). Recent studies assessing extended approaches, such as reboot surgery [15••], regenerative surgery (reboot surgery plus mucoplasty) [16••, 30], or extended full-house surgery [14], show that these approaches are likely to yield improved clinical and QoL outcomes compared to functional approaches in the more severe patients. However, these studies are qualified as having low levels of evidence due to the risk of bias related to the absence of placebo, non-randomization, and/or the high heterogeneity in their design [31].

The current different surgical techniques proposed thus far do not yet allow optimal disease management, submitting high polypoid recurrences [8, 32]. Even so, EESS has been proposed as a more effective approach to achieving lower revision rates [6, 13, 33]. The anatomical landmarks for more extended resections have already been emphasized [14, 24]. These approaches involve removing all nasal polyps and septa of the nasal and paranasal sinuses to achieve broad exposure while preserving the macroscopically healthy mucosa as a substrate for local healing. In contrast, newer approaches, such as reboot surgery [15••], focus on the newly argued hypothesis of the mucosal concept [9•], and propose resecting both the pathological and the surrounding healthy mucosa. This hypothesis assigns a transcendental role to the mucosal barrier in the inflammatory burden of CRSwNP pathophysiology, in which biomarkers contributing to polypoid recurrence have been identified [13, 34]. Mucoplasty as a regenerative surgery seeks to improve local control of the inflammatory burden after completely resecting the mucosa (reboot surgery) and by positioning a free mucosal graft from the floor of the nostril, which has different molecular and cellular features, with a lower tendency to polyp growth, adding these so-called regenerative properties [16••, 35]. Our group has conducted a new line of investigation to support the hypothesis of mucoplasty as a regenerative approach. The role of fibroblasts in the pathogenesis of CRSwNP, especially in the remodeling of the mucosa, is the basis for this hypothesis. In a recently published study, our group has shown that nasal polyp fibroblasts are a source of pro-inflammatory signaling that reinforce type 2 inflammation in CRSwNP [35].

Consequently, it could be targeted for therapeutic purposes as a potential novel source of inflammatory signaling [36]. Our experiments have also shown that the distribution of inflammatory cells differs in different locations of the nasal cavity, as well as in the polypoid mucosa and healthy mucosa [34]. This observed disparity may further strengthen the rationale for considering the nasal cavity floor mucosa as a suitable candidate for mucosal transplantation added to reboot surgery, arguing a better graft’s inflammatory and healing properties and supporting the concept of regenerative surgery. Additional tissue and molecular analyses of the inflammatory changes experienced by the mucosal graft after its placement in the ethmoidal roof are required to evaluate the functionality of these new findings and to strengthen the role of mucoplasty in sinonasal mucosal healing and healthy regeneration. More studies are needed to demonstrate the results of bilateral mucoplasty associated with reboot surgery in clinical practice, which will highlight this technique’s choice in managing severe CRSwNP and its recurrences.

The advances in the knowledge of the molecular basis of CRSwNP inflammation and the different related phenotypes have led to a precision medicine approach for diagnosing and managing CRSwNP patients [4, 11, 12]. There have been shown that patients who require revision surgeries present worse values for tissue, nasal secretion, and peripheral blood T2 phenotype biomarkers such as IL-5, IL-5 receptor alpha, and ECP [22]. Something similar was concluded concerning higher eosinophil counts in tissue and peripheral blood in patients with a tendency to polypoid recurrence [6, 37]. However, these findings seem controversial as they disappear in the long-term follow-up [21]. Studies assessing the efficacy of surgery in CRSwNP have underscored the need for biomarkers that define severe, uncontrolled, and recurrent disease to select the adequate surgical technique for each phenotype [12, 13, 38]. This enables the proposal for various extended approaches and complementary surgical procedures for patients exhibiting the more severe type 2 inflammatory endotype, such as reboot surgery adding mucoplasty, to improve their clinical outcomes and strengthen the paradigm of precision surgery [15••, 16••].

Results in our systematic review show that some extended approaches targeting the different lamellas and mucosa reach superior results in increasing QoL scores and symptom control in severe CRSwNP (Table 2). Our group has also shown that endonasal mucoplasty, complementary to reboot surgery, improves short- and medium-term outcomes versus isolated extended techniques (i.e., reboot surgery) [16••, 39]. First reports show that these mucosal grafts were used unilaterally with better local healing than the contralateral nostril in the short term [30, 39]. Subsequently, a prospective cohort study with bilateral mucoplasty showed hopeful outcomes in 54 patients with a type 2 inflammatory phenotype. An increase of up to 22.6 ± 6.3 units in the SNOT-22 QoL questionnaire was observed when using mucoplasty compared to patients who underwent only reboot surgery [16••]. These outcomes may be attributed to the early and sustained healing, facilitated by a regenerative process initiated by the mucosal autograft placed in the ethmoidal roof, with distinct cellular, molecular, and reparative features [35].

On the other hand, a significant decrease in polyp size was observed in all studies included in our systematic review, with a higher improvement in patients undergoing extended approaches, being consistent among published articles, with better average NPS values in medium- and long-term follow-up after EESS [16••, 20, 21], compared to worse results after functional surgeries [6, 26]. Recovery in polypoid size, edema, and nasal discharge have also been found by other authors with different extended approaches, such as complete [24] or full-house surgery [23], but not being significantly superior in extended versus functional ESS when analyzing endoscopic modified Lund Kennedy scale scores at short- or medium-term follow-up. Although significant enhancements in endoscopic scores were observed in EESS, no significant differences have been found among different types of surgeries in the long-term scores [6, Martin-Jimenez et al. - data not published]. The heterogeneity in study samples and the inaccurate surgical technique description may justify this variability. Moreover, post-surgical polypoid recurrences of over 40% have been reported, compared to 80–85% for edema at medium and long term [6, 23, 24, 32].

There has been shown that CRSwNP frequently appears alongside other respiratory inflammatory diseases such as asthma or N-ERD with a common underlying pathophysiology [40, 41]. The coincidence of bronchial and sinonasal type 2 inflammatory disease phenotype is a marker of poor control and higher severity for both diseases [11, 12]. Our group has recently reviewed a retrospective cohort of 274 patients with severe CRSwNP treated surgically with extended or functional ESS. We found an OR of 6.49 (95% CI 1.70, 24.84) for achieving an increase of at least 12 points in SNOT-22 scores comparing EESS vs FESS, and this improvement was independent of the asthmatic status of patients [Martin-Jimenez et al. - data not published]. DeConde et al*.* have already published a series of patients who underwent functional or extended ESS, finding no differences in surgical outcomes for CRSwNP in asthmatic vs no asthmatic patients [24]. This has also been shown in studies with longer-term follow-ups of CRSwNP patients treated surgically in which authors did not find differences in QoL outcomes in the subgroup of asthmatic patients compared to no asthmatic group [21]. These findings highlight the role of different types of surgery, indicating that surgical extension may lead to a significant role in achieving improved clinical and QoL outcomes, irrespective of comorbidities [6, 16••, 42].

New biologic drugs recently assessed for treating severe type 2 CRSwNP patients who do not achieve control even after surgery have been shown to address results in a way previously unattainable by medical and surgical therapy [43]. However, its cost-effectiveness still needs to be improved [44, 45]. A better efficiency related to ESS against biological drugs has been shown for achieving symptom control and improving QoL in the short- and medium-term [46–48]. In addition, experts groups, International Consensus, and Clinical Guidelines continue to advocate surgery before the use of these biological drugs [2–4, 7] and published results from clinical trials and real-life studies continue to demonstrate better outcomes in patients undergoing biological drugs after ESS versus those obtained by the treatments in isolation [49, 50]. However, surgical timing remains an ongoing topic of discussion.

## Limitations

The main limitation of our review is the impossibility of performing a meta-analysis of data, carrying out only a qualitative analysis, due to the high heterogeneity and low quality of the studies included. Our results are also limited by the need for a more scientific quality of the studies involving surgical procedures due to the nuances of each technique from an anatomical point of view, describing different extensions in the resection of the lamellae and the differences in the treatment of the nasal mucosa. This issue highlights the need for a consensual classification that allows us to accurately describe the action on the lamellae, the ostium, the extension in the resection of the septa, and the mucosa treatment.

Furthermore, the absence of randomized placebo-controlled studies, subject to the difficulties of using a placebo, and the ethical limitation of the indication of different techniques in patients with similar baseline characteristics inherent to surgery studies, implies that our results lack sufficient scientific evidence to be able to draw conclusions and draw up clinical guidelines.

Finally, the clinical outcomes associated with the use of mucoplasty in an environment where new advances in the cellular and molecular understanding of the inflammatory response in CRSwNP patients are modulating targeted therapies compel the development of a deeper line of investigation of the markers that define the peculiarities in the behavior of the nasal mucosa, thorough the different locations within the nasal fossa and paranasal sinuses. The new results associated with the study of fibroblasts and the other markers that modulate the inflammatory response may be the key to understanding the advantages of bilateral mucoplasty in treating severe CRSwNP.

## Conclusions

Our systematic review shows promising results for extended endoscopic sinus surgery with added mucoplasty in managing severe chronic rhinosinusitis with nasal polyp patients. The low quality of the current evidence found in this review does not allow to set robust recommendations about the most appropriate option for surgical treatment. There is a need for high-quality studies on phenotyping these patients to select those who will benefit more from each medical and surgical management option, including the combination of both, from a precision medicine point of view, including long-term efficiency as an essential outcome.

### Supplementary Information

Below is the link to the electronic supplementary material.

Supplementary file1 (PDF 190 KB)

## Data Availability

All the data and materials used in this publication are available in online databases. Those that required paper format are kept in the Otolaryngology Department of the Virgen Macarena University Hospital in Seville, Spain.
