# Supplementary material for: Endoscopic Extended Sinus Surgery for Patients with Severe Chronic Rhinosinusitis with Nasal Polyps, the Choice of Mucoplasty: A Systematic Review
Source: Curr Allergy Asthma Rep. 2023 Nov 22;23(12):733–46. doi: 10.1007/s11882-023-01113-x (PMC10739272; doi:10.1007/s11882-023-01113-x)
Supplement: Supplementary file 1 — Supplementary file1 (PDF 190 KB) [file 11882_2023_1113_MOESM1_ESM.pdf]

Supplementary Table S1. Types of ESS published to date. Thorough description of their characteristics, attending to each of the anatomic structures of sinonasal cavity.

	Functional Endoscopic Endonasal Surgery (FESS). Messerklinger, 1978; Kennedy, 1985; Stammberger, 1990	Nasalization (radical ethmoidectomy). Jankowski et al., 1995–2006	Full-house Functional Endoscopic Sinus Surgery (FHFESS). Seiberling et al., 2009	Reboot surgery. Alsharif et al., 2019	Regenerative surgery (reboot surgery plus mucoplasty). Moreno-Luna et al., 2019
**Rationale**	Conservative approach targeting osteomeatal complex disease	The most complete resection possible of the non-olfactory ethmoid mucosa from the underlying periosteum based on the evo-devo theory	To address the affected sinuses (computed tomography images), irrespective of the presence of specific sinus-related symptoms	Aims to maximally remove all sinus mucosa and enables healthy re-epithelialization from the preserved nasal mucosa to treat type 2 inflammation	To place mucosa from a non-polyposis donor site to promote re-epithelialization of the excised mucosa with a tissue of lesser polyposis potential
**Objective**	Removal of varying amounts of nasal and sinus respiratory epithelium to widen the natural sinus ostia to restore ventilation and mucociliary clearance of the nasal sinuses damage to the surrounding tissue	To perform a wide marsupialization of the ethmoid, maxillary, sphenoid, and frontal sinuses into the nasal cavities	Removal of all offending ethmoidal lamellae can prevent unintended obstruction and simplifies ongoing diagnostics and therapeutics in the postoperative patient	To accomplish a total clearance of all affected mucosa from all sinuses, leaving the periosteum where possible	To place mucosa from a non-polyposis donor site to promote re-epithelialization from the graft
**Mucosa**	Targeted removal of disease from key areas of the anterior ethmoid and middle meatus	Ethmoid mucosa complete removal. The mucosa on the walls of the large sinuses and around the frontal ostia is preserved	Targeted removal of disease from key areas of the anterior ethmoid and middle meatus	Complete removal	Complete removal
**Uncinectomy**	Performed systematically	Performed systematically	Performed systematically	Performed systematically	Performed systematically
**Ethmoidal bulla**	Once the cell walls are fractured, they are removed	Once the cell walls are fractured, they are removed	Once the cell walls are fractured, they are removed	Once the cell walls are fractured, they are removed	Circumferential dissection and complete removal
**Middle turbinate**	Preservation preferred	Systematically removed. Preserved (for mucosa medial side preservation) after technique updating	Consider middle turbinate medialization, or even securing the turbinate to the septum by inducing synechiae	Preserved as much as possible as a landmark, except for the parts that are destroyed or occupied by the disease or the anterior parts that need to be taken during the Draf III procedure	Preservation preferred
**Vertical plate of the basal lamella of the middle turbinate**	The basal lamella is perforated to enter the posterior ethmoid cells and the opening is enlarged	The basal lamella is perforated to enter the posterior ethmoid cells and the opening is enlarged	The basal lamella is perforated to enter the posterior ethmoid cells and the opening is enlarged	The basal lamella is perforated to enter the posterior ethmoid cells and the opening is enlarged	The basal lamella is perforated to enter the posterior ethmoid cells and the opening is enlarged
**Ethmoid bony lamellae**	On demand	Removed systematically	Removed systematically	Removed systematically	Removed systematically
**Middle meatal antrostomy**	On demand	Spared when possible	As large as possible	Wide antrostomy	Limited enlargement
**Maxillary sinus mucosa**	Removal of localized irreversible disease	The maxillary sinus membrane and ostium are conserved	On demand	Complete clearance	Complete clearance
**Ethmoid sinus mucosa**	On demand	Completely removed systematically	Removed systematically	Complete clearance, including the lamina orbitalis, skull base, and the lateral aspects of the middle turbinate	Complete clearance, including the lamina orbitalis, skull base, and the lateral aspects of the middle turbinate
**Sphenoidotomy**	On demand	Spared when possible	Wide	Wide	Wide
**Sphenoid sinus mucosa**	Preserved	On demand	Preserved unless grossly abnormal	Complete clearance	Complete clearance
**Frontal sinus opening**	Not intended	Ostial mucosa of each sinus is to be conserved if possible	Draf IIA, Draf III when indicated	Draf IIA, Draf III when indicated	Draf IIA, Draf IIB, Draf III
**Frontal sinus mucosa**	Preserved	Preserved	Frontal pathway clearance	Complete clearance	Removed as possible
**Adjunct procedures**			Canine fossa trephination and frontal minitrephination		Mucoplasty from nasal floor

Supplementary Table S2. PRISMA checklist fulfilled for this systematic review.

Section and Topic	Item #	Checklist item	Location where item is reported
**TITLE**			
Title	1	Identify the report as a systematic review.	Page 1
**ABSTRACT**			
Abstract	2	See the PRISMA 2020 for Abstracts checklist.	Page 1 (Lines 2-25)
**INTRODUCTION**			
Rationale	3	Describe the rationale for the review in the context of existing knowledge.	Pages 2-3 (Lines 28-65)
Objectives	4	Provide an explicit statement of the objective(s) or question(s) the review addresses.	Page 3 (Lines 66-69)
**METHODS**			
Eligibility criteria	5	Specify the inclusion and exclusion criteria for the review and how studies were grouped for the syntheses.	Page 5 (Lines 100-106)
Information sources	6	Specify all databases, registers, websites, organisations, reference lists and other sources searched or consulted to identify studies. Specify the date when each source was last searched or consulted.	Page 4 (Lines 90-92)
Search strategy	7	Present the full search strategies for all databases, registers and websites, including any filters and limits used.	Page 4 (Lines 82-98) and Table S3
Selection process	8	Specify the methods used to decide whether a study met the inclusion criteria of the review, including how many reviewers screened each record and each report retrieved, whether they worked independently, and if applicable, details of automation tools used in the process.	Page 5 (Lines 108-120)
Data collection process	9	Specify the methods used to collect data from reports, including how many reviewers collected data from each report, whether they worked independently, any processes for obtaining or confirming data from study investigators, and if applicable, details of automation tools used in the process.	Page 5 (Lines 108-120)
Data items	10a	List and define all outcomes for which data were sought. Specify whether all results that were compatible with each outcome domain in each study were sought (e.g. for all measures, time points, analyses), and if not, the methods used to decide which results to collect.	Page 5 (Lines 112-120)
	10b	List and define all other variables for which data were sought (e.g. participant and intervention characteristics, funding sources). Describe any assumptions made about any missing or unclear information.	Pages 5-6 (Lines 112-120) and Table S1
Study risk of bias assessment	11	Specify the methods used to assess risk of bias in the included studies, including details of the tool(s) used, how many reviewers assessed each study and whether they worked independently, and if applicable, details of automation tools used in the process.	Pages 5-6 (Lines 122-127), Figure 2 and Table S4
Effect measures	12	Specify for each outcome the effect measure(s) (e.g. risk ratio, mean difference) used in the synthesis or presentation of results.	Page 5 (Lines 112-120)
Synthesis methods	13a	Describe the processes used to decide which studies were eligible for each synthesis (e.g. tabulating the study intervention characteristics and comparing against the planned groups for each synthesis (item #5)).	Page 5 (Lines 100-111) and Table S3
	13b	Describe any methods required to prepare the data for presentation or synthesis, such as handling of missing summary statistics, or data conversions.	Page 6 (Lines 129-142)
	13c	Describe any methods used to tabulate or visually display results of individual studies and syntheses.	Page 6 (129-133)
	13d	Describe any methods used to synthesize results and provide a rationale for the choice(s). If meta-analysis was performed, describe the model(s), method(s) to identify the presence and extent of statistical heterogeneity, and software package(s) used.	Page 6 (129-133)
	13e	Describe any methods used to explore possible causes of heterogeneity among study results (e.g. subgroup analysis, meta-regression).	Not applicable
	13f	Describe any sensitivity analyses conducted to assess robustness of the synthesized results.	Not applicable
Reporting bias assessment	14	Describe any methods used to assess risk of bias due to missing results in a synthesis (arising from reporting biases).	Pages 5-6 (Lines 122-127)
Certainty assessment	15	Describe any methods used to assess certainty (or confidence) in the body of evidence for an outcome.	Figure 2 and Table S4

Supplementary Table S2. PRISMA checklist fulfilled for this systematic review (continue).

Section and Topic	Item #	Checklist item	Location where item is reported
**RESULTS**			
Study selection	16a	Describe the results of the search and selection process, from the number of records identified in the search to the number of studies included in the review, ideally using a flow diagram.	Figure 1
	16b	Cite studies that might appear to meet the inclusion criteria, but which were excluded, and explain why they were excluded.	Page 5 (100-106) Figure 1 and Table S3
Study characteristics	17	Cite each included study and present its characteristics.	Tables 1 and 2
Risk of bias in studies	18	Present assessments of risk of bias for each included study.	Figure 2 and Table S4
Results of individual studies	19	For all outcomes, present, for each study: (a) summary statistics for each group (where appropriate) and (b) an effect estimate and its precision (e.g. confidence/credible interval), ideally using structured tables or plots.	Table 2
Results of syntheses	20a	For each synthesis, briefly summarise the characteristics and risk of bias among contributing studies.	Table 1, Figure 2 and Table S4
	20b	Present results of all statistical syntheses conducted. If meta-analysis was done, present for each the summary estimate and its precision (e.g. confidence/credible interval) and measures of statistical heterogeneity. If comparing groups, describe the direction of the effect.	Not applicable
	20c	Present results of all investigations of possible causes of heterogeneity among study results.	Tables 1 and 2
	20d	Present results of all sensitivity analyses conducted to assess the robustness of the synthesized results.	Not applicable
Reporting biases	21	Present assessments of risk of bias due to missing results (arising from reporting biases) for each synthesis assessed.	Not applicable
Certainty of evidence	22	Present assessments of certainty (or confidence) in the body of evidence for each outcome assessed.	Page 6 (Lines 130-145), Figures 1-2, and Table S4
**DISCUSSION**			
Discussion	23a	Provide a general interpretation of the results in the context of other evidence.	Page 13 (Lines 183-203),
	23b	Discuss any limitations of the evidence included in the review.	Pages 18-19 (Lines 320-329)
	23c	Discuss any limitations of the review processes used.	Pages 18-19 (Lines 320-343)
	23d	Discuss implications of the results for practice, policy, and future research.	Pages 13-19 (204-318)
**OTHER INFORMATION**			
Registration and protocol	24a	Provide registration information for the review, including register name and registration number, or state that the review was not registered.	Page 3 (Lines 73-74)
	24b	Indicate where the review protocol can be accessed, or state that a protocol was not prepared.	Page 4 (Lines 82-98) and Table S3
	24c	Describe and explain any amendments to information provided at registration or in the protocol.	Not applicable
Support	25	Describe sources of financial or non-financial support for the review, and the role of the funders or sponsors in the review.	Title page
Competing interests	26	Declare any competing interests of review authors.	Title page
Availability of data, code and other materials	27	Report which of the following are publicly available and where they can be found: template data collection forms; data extracted from included studies; data used for all analyses; analytic code; any other materials used in the review.	Table 2 and Table S3

Supplementary Table S3. Search strategy.

**Researchers**	Three authors performed the screening by title and abstract (DMJ, RML, JMS)
**Databases used**	PubMed, The Cochrane Library for Cochrane Reviews, Embase via Elsevier, Web of Science, and Scopus
**Medical Subject Headings (MeSH) terms**	('chronic rhinosinusitis with nasal polyps' OR 'nasal polyps' OR 'nasal polyposis' OR 'crswnp' OR 'nose polyp' OR 'nasal cavity polyp' OR 'nose polyposis') AND ('sinus surgery' OR 'endoscopic sinus surgery' OR 'ess' OR 'extended endoscopic sinus surgery' OR 'full house surgery' OR 'reboot surgery' OR 'complete surgery' OR 'nasalization' OR 'surgery') NOT ('monoclonal antibody' OR 'monoclonal antibodies' OR 'monoclonal drug' OR 'biologic therapy' OR 'biologic drug' OR 'biologics')
**Filters applied**	• Text availability: abstract and/or full text<br>• Article types: clinical trials, cohort studies, case-control studies, cross-sectional studies or case-series studies (previous meta-analysis, systematic reviews, case reports and books chapters were excluded)<br>• Article language: English<br>• Species: Humans
**Inclusion criteria**	**Participants**: Severe CRSwNP patients older than 18, non-responders to adequate medical treatment who undergo ESS.<br>**Intervention**: Eligible interventions included extended ESS (e.g., nasalization, complete, radical, full-house, reboot, reboot) with or without mucoplasty.<br>**Comparators**: Standard of care medical or surgical treatment.<br>**Outcomes**: Quality of life improvement, symptom scale improvement, changes in nasal endoscopy scores, and/or Computed Tomography (CT) scan scores.<br>**Timing and Setting**: With no limitations.
**Studies found in each database prior to screening by the authors**	• EMBASE via Elsevier = 292 studies<br>• Pubmed = 275 studies<br>• Web of Science = 233 studies<br>• Cochrane library = 176 studies<br>• Scopus = 23 studies
**Studies included in systematic review after inclusion and exclusion criteria were implemented**	• 13 studies of scientific literature<br>• An unpublished article of its own was added and analyzed in this review, getting a total of 14 studies

Supplementary Table S4. Quality Assessment of case series studies checklist from the National Institute for Health and Clinical Excellence (Appendix F) applied to this systematic review.

	1.1	1.2	1.3	2.1	2.2	2.3	2.4	2.5	2.6	2.7	2.8	2.9	2.10	3.1	3.2	3.3	3.4	3.5	3.6	4.1	4.2	4.3	4.4	4.5	4.6	5.1	5.2
**Batra *et al*, 2003**	+	-	-	NA	NA	NA	-	+	NA	NA	++	+	+	+	+	+	+	NA	+	NA	NA	-	-	-	-	+	-
**Jankowski *et al*, 2006**	+	+	+	-	+	NA	-	+	NA	-	+	+	+	+	+	+	+	-	+	NR	NA	-	-	+	+	+	+
**Proimos *et al*, 2010**	++	+	+	NA	NA	NA	-	+	NA	NA	-	+	+	+	++	++	+	NA	+	NA	NA	-	-	+	+	+	+
**Shen *et al*, 2011**	++	+	+	NA	NA	NA	-	+	NA	NA	-	++	++	+	+	+	+	NA	+	NA	NA	-	-	+	-	+	+
**Zhang *et al*, 2014**	+	+	+	-	+	NA	-	+	NA	-	+	+	+	+	++	++	+	++	+	-	NA	+	-	+	+	+	+
**DeConde *et al*, 2015**	++	++	+	-	+	NA	-	+	NA	+	-	+	++	+	++	+	+	+	+	++	NA	-	-	++	++	+	+
**Chen *et al*, 2016**	++	+	+	+	++	NA	-	+	NA	+	++	+	+	+	++	++	++	++	+	+	NA	+	-	+	+	+	+
**Alsharif *et al*, 2019**	++	+	+	NA	+	NA	-	+	NA	+	-	+	+	+	+	+	+	+	+	+	NA	-	-	-	+	+	+
**Calus *et al*, 2019**	++	+	+	NA	+	NA	-	+	NA	NA	+	+	+	++	+	+	+	NA	++	NA	NA	-	-	+	+	+	+
**Zhang *et al*, 2020**	+	+	+	-	++	NA	-	+	NA	++	++	++	+	+	+	+	++	++	++	++	NA	++	+	++	+	++	+
**Arancibia *et al*, 2022**	-	+	+	NA	NA	NA	-	+	NA	NA	-	+	+	+	+	+	+	NA	++	NA	NA	-	-	+	+	+	+
**Moreno-Luna *et al*, 2022**	++	++	+	+	++	NA	-	++	NA	++	++	+	+	++	++	++	++	++	+	++	NA	-	+	++	++	++	+
**Pirola *et al*, 2022**	++	+	+	NA	+	NA	-	+	NA	++	++	+	+	+	++	+	++	+	+	-	NA	+	+	+	+	+	+
**Martin-Jimenez *et al*, NP***	++	+	+	-	+	NA	-	+	NA	++	++	++	+	+	++	+	+	++	+	++	NA	-	+	++	++	++	+

Questions of Appendix F → Section 1: Population. 1.1 Is the source population or source area well described? **1.2** Is the eligible population or area representative of the source population or area? **1.3** Do the selected participants or areas represent the eligible population or area? **Section 2: Method of allocation to intervention (or comparison). 2.1** Allocation to intervention (or comparison). How was selection bias minimised? **2.2** Were interventions (and comparisons) well described and appropriate? **2.3** Was the allocation concealed? **2.4** Were participants or investigators blind to exposure and comparison? **2.5** Was the exposure to the intervention and comparison adequate? **2.6** Was contamination acceptably low? **2.7** Were other interventions similar in both groups? **2.8** Were all participants accounted for at study conclusion? **2.9** Did the setting reflect usual UK practice? **2.10** Did the intervention or control comparison reflect usual UK practice? **Section 3: Outcomes. 3.1** Were outcome measures reliable? **3.2** Were all outcome measurements complete? **3.3** Were all important outcomes assessed? **3.4** Were outcomes relevant? **3.5** Were there similar follow-up times in exposure and comparison groups? **3.6** Was follow-up time meaningful? **Section 4: Analyses. 4.1** Were exposure and comparison groups similar at baseline? If not, were these adjusted? **4.2** Was intention to treat (ITT) analysis conducted? **4.3** Was the study sufficiently powered to detect an intervention effect (if one exists)? **4.4** Were the estimates of effect size given or calculable? **4.5** Were the analytical methods appropriate? **4.6** Was the precision of intervention effects given or calculable? Were they meaningful? **Section 5: Summary. 5.1** Are the study results internally valid (i.e. unbiased)? **5.2** Are the findings generalisable to the source population (i.e. externally valid)?

++ Indicates that for that particular aspect of study design, the study has been designed or conducted in such a way as to minimise the risk of bias. **+** Indicates that either the answer to the checklist question is not clear from the way the study is reported, or that the study may not have addressed all potential sources of bias for that particular aspect of study design. **–** Should be reserved for those aspects of the study design in which significant sources of bias may persist. **NR (not reported)** Should be reserved for those aspects in which the study under review fails to report how they have (or might have) been considered. **NA (not applicable)** Should be reserved for those study design aspects that are not applicable given the study design under review (for example, allocation concealment would not be applicable for case control studies).

***NP** = Not published.
